# Hyaluronic Acid Enriched Medium for Embryo Transfer: A Systematic Review and Meta-analysis

**DOI:** 10.5935/1518-0557.20260012

**Published:** 2026

**Authors:** Rodrigo Manieri Rocha, Maira C Ribeiro Andrade, Ionara Diniz Evangelista Santos Barcelos, Iara Gonçalves Roberto Viana, Wellington de Paula Martins

**Affiliations:** 1 SEMEAR Fertilidade - Human Assisted Reproduction. Ribeirão Preto, São Paulo, Brazil; 2 Uberlandia Medical Center - Endometriosis, Advanced Gynecological Ultrasound and Minimally Invasive Surgery Division, Uberlândia, Minas Gerais, Brazil; 3 Uberlandia Medical Center - Human Assisted Reproduction Division, Uberlândia, Minas Gerais, Brazil; 4 Federal University of Uberlândia, Uberlândia, Minas Gerais, Brazil

**Keywords:** hyaluronic acid, hyaluronan, embryoglue, embryo transfer, assisted reproduction

## Abstract

This systematic review and meta-analysis of randomized controlled trials (RCTs) evaluated whether the use of hyaluronic acid (HA)-enriched medium for embryo transfer (ET) improves live birth rates in in-vitro fertilization (IVF) cycles. A comprehensive search of PubMed, Scopus, Web of Science, and ClinicalTrials.gov was conducted up to December 2024, without language restrictions, and only RCTs comparing HA-enriched to standard ET media were included. Risk of bias was assessed using the Cochrane tool, meta-analyses were performed using a random-effects model with the Mantel-Haenszel method, sensitivity analyses excluded studies at high risk of bias, and evidence quality was evaluated with GRADE. Of 450 records identified, 24 studies were eligible, of which 20 were included in the meta-analysis, and most studies had a high risk of bias. Eleven studies reported live birth outcomes, with 2,026 versus 2,037 participants and 803 versus 697 live births in the HA-enriched and standard medium groups, respectively. The relative risk (RR) for live birth was 1.14 (95% CI: 0.99-1.31; *p*=0.07), with moderate heterogeneity (I^2^=54%), and sensitivity analysis showed an RR of 1.06 (95% CI: 0.85-1.31; *p*=0.62). Clinical pregnancy rates were initially higher with HA-enriched medium (RR=1.17; 95% CI: 1.05-1.29; *p*=0.004), but this effect disappeared in sensitivity analysis (RR=1.05; *p*=0.53), and pregnancy loss showed no significant difference between groups. Previous studies had suggested a potential benefit of HA-enriched ET medium on live birth outcomes; however, in the present analysis no significant difference in live birth rates was observed between HA-enriched and standard ET media. Overall evidence quality was rated as very low due to high risk of bias, inconsistency, and imprecision, and current evidence does not support a clear benefit of HA-enriched ET medium, although ongoing trials may further clarify its role.

## INTRODUCTION

Uterine embryo implantation after transfer is a key step in assisted reproduction treatments. For instance, in Europe in 2019, the mean pregnancy rate per embryo transfer was 34.6% after IVF, 32.1% after ICSI, 35.9% after frozen embryo transfer and 50.5% after egg donation (European IVF Monitoring Consortium (EIM) for the European Society of Human Reproduction and Embryology (ESHRE) *et al.*, 2023). The mechanisms that explain successes and failures are still poorly understood. However, many alternatives were proposed to potentially increase the number of successful implantations, and Hyaluronic acid (HA) is frequently used to possibly improve the reproductive results (Holt-Kentwell *et al.*, 2022). HA is a high molecular weight glycosaminoglycan of the extracellular matrix without antigenic properties, and it is essential for creating and filling extracellular spaces, being naturally present in almost all body tissues, including the genital tract (Rodriguez-Martinez *et al.*, 2016). The early *in vitro* studies using a mouse model proposed that HA promotes cell to cell and cell to matrix adhesions via its receptor CD44, which is expressed on the preimplantation embryo and also on the endometrial stroma in mammals (Cowman *et al.*, 2015; Adeniyi *et al.*, 2021). HA is used in embryo transfer medium because it increases its viscosity without imposing any harm to the embryo (Cowman *et al.*, 2015). Additionally, it is thought that HA can facilitate implantation by promoting cellular interactions (Adeniyi *et al.*, 2021), and the HA-enriched medium for embryo transfer is a commercially available add-on for assisted reproductive technology (ART) (Lensen *et al.*, 2021).

In the last two decades, several studies have evaluated the effectiveness of HA-enriched medium for embryo transfer (Valojerdi *et al.*, 2006; Urman *et al.*, 2008; Fancsovits *et al.*, 2015), and there are also some recent systematic reviews on this topic (Heymann *et al.*, 2020; 2022; Tyler *et al.*, 2022). All these three systematic reviews concluded that HA-enriched medium might improve reproductive outcomes, and the quality of the evidence was judged to be moderate (Heymann *et al.*, 2022). However, since the publication of these reviews, new RCTs with considerable sample sizes have been published, showing no beneficial effect of this intervention (Yung *et al.*, 2021), and there is also evidence that infusion of the uterine cavity with HA might actually worsen the endometrial receptivity (Marei *et al.*, 2017).

The objective of this systematic review is to identify, assess, and summarize evidence on the effectiveness and safety of HA-enriched medium compared with the standard medium for embryo transfer.

## METHODS

### Registration

The protocol of this review was registered at PROSPERO under CRD42024579125, available at https://www.crd.york.ac.uk/prospero/display_record.php?ID=CRD42024579125

### Information sources

Published and unpublished studies were searched, without language restriction, in PubMed, Scopus, Web of Science and Clinicaltrials.gov, until December 2024. Additionally, we hand-searched the reference list of the previously published systematic reviews on this topic.

### Search strategy

The following terms were used for the searches: (Hyaluronic OR hyaluronan OR Embryoglue OR Glue) AND (embryo OR IVF) AND (Random* OR Trial).

### Eligibility criteria

Regarding the study design, only randomized controlled trials were considered eligible. Crossover trials were considered eligible, but only data from the first phase were included in the quantitative analysis, as the crossover is not a valid design in this context. Quasi-randomized trials (allocation based on date of birth, day of the week, alternated) were not considered eligible. The participants were all women undergoing embryo transfer. The intervention in question used an enriched medium in comparison to a standard medium (that might contain lower concentrations of HA) for embryo transfer.

### Outcomes

The primary outcome is live birth per allocated woman. Ongoing pregnancy would be used as a surrogate for live birth when only the latter was available (Braakhekke *et al.*, 2014).

The secondary outcomes were clinical pregnancy per allocated woman and pregnancy loss per clinical pregnancy. Multiple pregnancies and the birth of twins were counted as single events.

Pregnancy loss was evaluated using clinical pregnancy as the denominator to avoid confusion when interpreting the results. For example, assume these theoretical results: Group 1=100 women, 60 clinical pregnancies, 12 pregnancy losses, 48 live births; Group 2=100 women, 30 clinical pregnancies, six pregnancy losses, 24 live births. By using the number of randomized women as the denominator, one should conclude that the risk of pregnancy loss is higher in group 1 (12% *vs*. 6%, groups 1 and 2, respectively). However, by using clinical pregnancy as the denominator, one would conclude that the pregnancy loss by clinical pregnancy is similar between groups (20% *vs*. 20%). We believe the latter makes more sense, avoiding the conclusion that the risk of pregnancy loss is greater in group 1 while the pregnancy loss by clinical pregnancy was precisely the same, and the observed difference only occurred because there were more clinical pregnancies in group 1.

### Selection process

Two reviewers (RMR and MCRA) read the title/abstracts of all records after excluding duplicates. All records that were considered potentially eligible by at least one of the reviewers were selected for evaluating the full text. The next step was performed by the same reviewers, reading the entire manuscript of the records that were selected in the first phase to evaluate whether the study was eligible or not. Disagreements were solved by consulting another reviewer (WPM).

### Data collection process

Two reviewers (RMR and MCRA) independently extracted data from the eligible studies. Additionally, other reviewer (WPM) compared the extract data with those reported in previously published systematic reviews. Disagreements were solved by discussion.

The following outcomes and data items were assessed: Live birth, ongoing pregnancy, clinical pregnancy and pregnancy loss.

We also evaluate the mean age (differences greater than 1.0 years were considered to be relevant), ovarian antral follicle count (differences greater than 2.0 follicles were considered to be relevant), anti-mullerian hormone AMH (differences greater than 0.5 ng/mL were supposed to be appropriate), and the number of transferred embryos (differences greater than 0.2 embryos were considered to be relevant). If age or the number of embryos transferred were not reported, the study was considered to be at high risk of bias.

Additionally, we assessed whether the embryo transfer medium in the control group had HA on its composition, and we performed a subgroup analysis separating the studies by this criterion.

### Study risk of bias assessment

Two reviewers (RMR and MCRA) independently evaluated the risk of bias of the included studies by using the Cochrane risk-of-bias tool for randomized trials version 2 (RoB 2) (Higgins *et al.*, 2024). Disagreements were solved by discussion with a third author (WPM).

### Effect measures

We assessed the risk ratios and their respective 95% confidence interval (CI).

### Synthesis methods

Data were combined for meta-analysis using Review Manager 5.4 using the Mantel-Haenszel method and a random-effects model. Heterogeneity was assessed by I^2^ statistics. Forest-plots were produced to summarize the analyses, and a sensitivity analysis was performed excluding the studies deemed at high-risk of bias.

### Reporting bias

When the one of the evaluated outcomes could not be extracted from the full texts, we evaluated whether the outcomes were reported in the published protocol clinicaltrials.gov. When the results were not available, we tried to contact the study authors to provide additional information. Additionally, we assessed the funnel-plot to evaluate the risk of reporting bias.

### Certainty assessment

We evaluate the quality/certainty of the evidence as suggested GRADE working group (https://www.gradeworkinggroup.org/), evaluating the limitations of the included studies, inconsistency, indirectness, imprecision, and publication bias. An evidence table was created reporting the judgements about evidence quality (high, moderate, low or very-low) with the justifications (Schünemann *et al.*, 2023).

### Artificial Intelligence Use Declaration

Large Language Models tools were used to English scientific language correction and readability improvement.

## RESULTS

### Search Results

**Figure 1:**
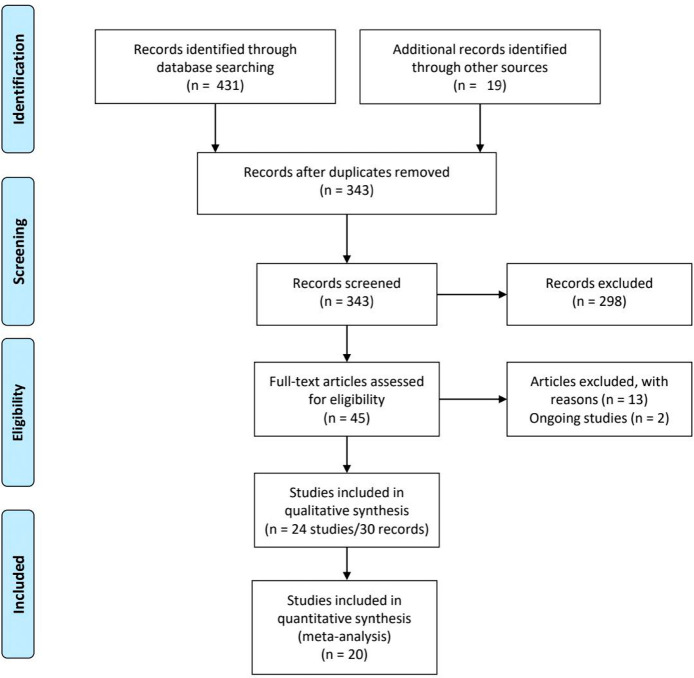
Search results.

The search results are reported in Figure 1. The last electronic search was performed in 2024.12.01 and retrieved a total of 431 records: PubMed=128; Scopus=125; Web of Science=160; and Clinicaltrials.gov=18. Nineteen additional records were added by manual search, and 107 duplicates were removed. A total of 343 records were screened based on title/abstracts, and 301 were excluded. A total of 45 records were completely assessed for eligibility: 13 records were excluded for some reason (Table 1), and we identified three ongoing trials (Cai *et al.*, 2025; Nogueira, 2025; Warhade *et al.*, 2025). A total of 24 studies (from 30 records) were considered eligible (Table 2). Two records (Ten *et al.*, 2019; Sellers *et al.*, 2022) reported the same cohort with separated randomization and different control groups; two studies had three records each, and the other two studies had two records each (Table 3).

**Table 1:** Excluded studies with reasons.

Study	Reason
Ben-Rafael *et al.,* 1995	A fibrin sealant enriched medium was used for embryo transfer in the intervention group.
Chao *et al.,* 2008	Quasi-randomized. Allocation based on the consecutive participant list.
Hambiliki *et al.,* 2010	Quasi-randomized. Allocating based on alternating weeks.
Karimian *et al.,* 2004	Additional reference to Valojerdi *et al.* (2006).
Kleijkers *et al.,* 2016	HA enriched medium was not tested, Instead, authors have randomly assigned the participants to have their oocytes and embryos cultured in one of the two media: G5 or HTF.
Nakagawa *et al.,* 2012	Quasi-randomized. Allocation based on odd or even ID numbers.
Nakagawa *et al.,* 2023	Prospective observational study.
Nishihara & Morimoto, 2017	Quasi-randomized. Allocation based on the days of the week.
Perez *et al.,* 2019	Quasi-randomized trial. Allocating based on ID number.
Thornton *et al.,* 2018	Not randomized (case control study).
Tomari *et al.,* 2014	Quasi-randomized. Allocation based on the days of the week.
Valojerdi *et al.,* 2006	Quasi-randomized. Allocation based on alternating days.

**Table 2:** Included studies

Study	Country/Center	Enrolment Period	Embryos	Intervention	Control
Balaban *et al.*, 2004	Turkey, American Hospital of Istanbul	2006.Jun to 2007.Feb	Fresh Blastocyst	N=193, Embryoglue (30 min)	N=193, G2 + HSA
Chen *et al.*, 2001	Taiwan, IVF-Unit, Dr. Tsai & Dr. Chen’s Women Hospital	Unclear	Unclear	N=35, HSA + HA	N=35, HSA
Child *et al.*, 2021	United Kingdom, TFP Oxford Fertility, Oxford	2017 to 2019	Fresh or frozen transfer of 1-2 cleavage stage embryos or blastocysts.	N=343 Embryoglue (10-30min)	N=338 COOK culture media
Dittmann-Müller *et al.*, 2009	Chemnitz, Germany, Women’s Hospital, IVF, Basel, Switzerland, Fertilitas, IVF/ICSI Centre, Würzburg, Germany, University Hospital Würzburg, Dept. of Obstetrics and Gynecolgy	2006.Jan to 2007.Mar	Fresh D3 embryos	N=54, EmbryoGlue (30min)	N=48, G-1 and G-2 version 3 plus + 10% HSA
Drew *et al.*, 2014	Unclear	Unclear	D3 or D5 embryos, single or double	N=Unclear EmbryoGlue (10min)	N=Unclear Standard media
Fancsovits *et al.*, 2015	Budapest, Hungary, Division of Assisted Reproduction, Semmelweis University	2010.Jan to 2012.Aug	D2 ou D3, fresh embryos	N=290, EmbryoGlue (10min)	N=291, G-2^+^ (Vitrolife)
Fasano *et al.*, 2016	Brussels, Belgium Hôpital Erasme, ULB, FIV Laboratoire	2015.Jun to 2016.Jan	Day2/Day3 and Day5/Day6	N=136 Embryo-Glue	N=192 Routine ET medium
Friedler *et al.*, 2005	Tel Aviv, Israel, IVF and Infertility Unit, Assaf Harofeh Medical Center Tel Aviv University	Unclear	D2 Embryos	N=94, Embryo-Glue	N=93, HTF medium + 20% SSS
Friedler *et al.*, 2007	Israel, Assaf Harofeh Medical Center	2005.Jun to 2006.Nov	D2 ou D3, fresh embryos	N=51, EmbryoGlue	N=50, HTF medium with gentamicin + 20% SSS
Hazlett *et al.*, 2008	Elgin, IL, Sherman Hospital	Unclear	D3 or D5 fresh embryos	D3=85 D5=32 EmbryoGlue (10-60min)	D3=79 D5=29 D3=IVC-1 or IVC-2 + 0.5% HSA D5= G2.3 + 0.5% HSA
Kandari, 2019	Mumbai, India, Cellsure Biotech and Research Centre	2017.Jan to 2018.Aug	Cleavage stage or blastocyst single fresh	N=153, EmbryoGlue	N=168, CSCM medium
Khan *et al.*, 2004	Michigan, USA, IVF Michigan, Rochester Hills, IVF and Andrology Laboratory	Unclear	D3 fresh embryos	N=Unclear EmbryoGlue (10min)	N=Unclear P1 Complete medium
Korosec *et al.*, 2007	Ljubljana, Slovenia, Department of Obstetrics and Gynecology, University Medical Centre Ljubljana. Bregenz, Austria, Institute for Reproductive Medicine and Endocrinology, Roemerstrasse	2004.Apr to 2006.Jun	Fresh and frozen single blastocyst transfer	N=28 (fresh) N=102 (frozen) EmbryoGlue	N=37 (fresh) N=112 (frozen) M2 medium
Mahani & Davar, 2007	Yazd, Islamic Republic of Iran, Research and Clinical Centre for Infertilityin	2003.Set to 2004.Jan	D3 Fresh embryos	N=30 EmbryoGlue (10min)	N=30 Medium containing albumin 20%
Morbeck, 2007	Mayo Clinic, Rochester, Minnesota, USA	2003.May to 2005.Jun	Frozen embryo transfers	N=41 Embryoglue	N=42 Standard medium
Ravhon *et al.*, 2005	Israel, The Edith Wolfson Medical center	2004.Jul to 2004.Nov	Fresh embryo transfers	N=79 EmbryoGlue (10min)	N=69 Medium G-1
Schoolcraft *et al.*, 2002	Englewood, Colorado; Colorado Ctr for Reproductive Medicine	2001.Jan to 2002.Feb	D3 fresh embryos	N=91 EmbryoGlue	N=84 Medium G2
Ten *et al.*, 2019	Alicante, Spain, Department of Reproductive Medicine, Instituto Bernabeu	2018.Mar to 2019.Jan	D5 Fresh single embryo transfer	N=48 (first randomization) EmbryoGlue	N=61 (first randomization) Global Total (GT)^®^, LifeGlobal^®^
Sellers *et al.*, 2022	Alicante, Spain, Department of Reproductive Medicine, Instituto Bernabeu	2018.Mar to 2019.Jan	D5 Fresh single embryo transfer	N=104 (second randomization) EmbryoGlue	N=89 (second randomization) Continuous Single Culture^®^ Complete (CSCC)
Simon *et al.*, 2003	Jerusalem, Israel, IVF unit, Departament of Obstretics and Gynecology, Hadassah University Hospital Ein Kerem	Unclear	D3 Fresh embryos	N=40, P1 medium supplemented with 0.5 mg/ml of highly purified fermentation-based hyaluronic acid (5-10min)	N=40, P1 Medium containing 10%of SSS
Urman *et al.*, 2008	Istanbul, Turkey, Assisted Reproduction Unit, American Hospital of Istanbul	2006.Jun and 2007.Feb	D3 or D5 fresh embryos	D3=412 D5=227 EmbryoGlue (30min)	D3=413 D5=230 G2, version 3 + HSA
Walker *et al.,* 2005	Rochester, MN, Mayo Clinic College of Medicine, London, United Kingdom, London Fertility Centre	Unclear	Frozen clevage embryo transfer	N=34 EmbryoGlue^®^	N=34 G1.3 media
Yakin *et al.,* 2001	Istanbul, Turkey, VKV American Hospital, Assisted Reproduction Unit	Unclear	Frozen-thawed embryo	N=65, EmbryoGlue	N=64, G2 culture medium
Yung *et al.*, 2021	Hong Kong The Centre of Assisted Reproduction and Embryology, The University of Hong Kong -Queen Mary Hospital, The Dr. Stephen Chow Chun-kay Assisted Reproduction Centre, Kwong Wah Hospital	2016. Apr to 2018.Apr	Frozen Cleavage stage embryos or blastocysts	N=275 EmbryoGlue	N=275 G-2 medium (Vitrolife)

HSA=Human serum albumin solution; HA=Hyaluronic Acid; IVC=In VitroCare; SSS= Synthetic serum substitute; HTF=human tubal fluid

**Table 3:** Studies with more than one record.

Study ID	Records	Notes
Fancsovits *et al.*, 2015	Fancsovits *et al.*, 2015	Full text published in Arch Gynecol Obstet
	Fancsovits, 2014	Registration in clincaltrials.gov, NCT02077608, however it was registered on Feb.28.2014, long after the study has finished the enrolment.
	Fancsovits *et al.*, 2011	Included in the Cochrane review as a different study. However, the enrolment period was Jan.2010 to Dec.2010 while the enrolment period of Fancsovits *et al.* (2015) was Jan.2010 and Aug.2012
Hazlett *et al.*, 2008	Hazlett *et al.*, 2008	Full text published as correspondence in Fertil Steril
	Hazlett *et al.*, 2004	Abstract of the 20th Annual Meeting of the ESHRE
Ten *et al.*, 2019; Sellers *et al.*, 2022	Sellers *et al.*, 2022	Abstract of the 2022 Conference of ALPHA Scientists in Reproductive Medicine
	Ten *et al.*, 2019	Abstracts of the 35th Annual Meeting of the ESHRE
Urman *et al.*, 2008	Urman *et al.*, 2008	Full text published in Fertil Steril
	Balaban *et al.*, 2011	Abstracts of the 27^th^ Annual Meeting of ESHRE
Yung *et al.,* 2021	Yung *et al.,* 2021	Full text published in Fertil Steril
	Yung *et al.*, 2019	Abstracts of the 35^th^ Annual Meeting of the ESHRE
	Yung, 2016	Registration in clincaltrials.gov NCT02725827

We extracted data from a previously published systematic review (Heymann *et al.*, 2022) for three studies: all data for 2 studies (Korosec *et al.*, 2007) and only data for live birth for the other study . We were not able to extract data from the outcomes of interest from 4 studies (Chen *et al.*, 2001; Khan *et al.*, 2004; Drew *et al.*, 2014; Fasano *et al.*, 2016) therefore, we included 20 studies in the quantitative analysis.

### Risk of bias results

The risk of bias assessment of the 24 included studies is reported on Table 4. One study (Yung *et al.*, 2021) was considered to have low risk of bias and we have some concerns regarding the risk of bias for another study (Hazlett *et al.*, 2008). The remaining 22 studies were deemed to be at high risk of bias.

**Table 4:** Risk of bias of the included studies

Study	Randomization Process	Deviations from the intended interventions	Missing outcome data	Measurement of the outcome	Selection of the reported result	Overall	Notes
Balaban *et al.*, 2004	High risk	High risk	High risk	High risk	High risk	High risk	A, B
Chen *et al.*, 2001	High risk	High risk	High risk	High risk	High risk	High risk	A
Child *et al.*, 2021	High risk	High risk	High risk	High risk	High risk	High risk	A
Dittmann-Müller *et al.*, 2009	High risk	High risk	High risk	High risk	High risk	High risk	A
Drew *et al.*, 2014	High risk	High risk	High risk	High risk	High risk	High risk	A
Fancsovits *et al.*, 2015	High risk	Some concerns	Some concerns	Some concerns	Some concerns	High risk	C
Fasano *et al.,* 2016	High risk	High risk	High risk	High risk	High risk	High risk	A, D
Friedler *et al.*, 2005	High risk	High risk	High risk	High risk	High risk	High risk	A
Friedler *et al.*, 2007	High risk	High risk	Some concerns	Some concerns	Some concerns	High risk	E
Hazlett *et al.*, 2008	Low risk	Some concerns	Some concerns	Some concerns	Some concerns	Some concerns	F
Kandari, 2019	High risk	High risk	High risk	High risk	High risk	High risk	A
Khan *et al.,* 2004	High risk	High risk	High risk	High risk	High risk	High risk	A
Korosec *et al.*, 2007	High risk	Some concerns	Some concerns	Some concerns	Some concerns	High risk	G
Mahani & Davar, 2007	High-risk	Some concerns	Some concerns	Some concerns	Some concerns	High risk	H
Morbeck, 2007	High risk	High risk	High risk	High risk	High risk	High risk	I
Ravhon *et al.*, 2005	High risk	High risk	High risk	High risk	High risk	High risk	A
Schoolcraft *et al.*, 2002	High risk	High risk	High risk	High risk	High risk	High risk	A
Sellers *et al.*, 2022	High risk	High risk	High risk	High risk	High risk	High risk	A, J
Simon *et al.*, 2003	High risk	Some concerns	Some concerns	Some concerns	Some concerns	High risk	K
Ten *et al.*, 2019	High risk	High risk	High risk	High risk	High risk	High risk	A, J
Urman *et al.*, 2008	Low risk	High risk	Low risk	Low risk	Some concerns	High risk	L
Walker *et al.* (2005)	High risk	High risk	High risk	High risk	High risk	High risk	A, M
Yakin *et al.* (2001)	High risk	High risk	High risk	High risk	High risk	High risk	A, N
Yung *et al.* (2021)	Low risk	Low risk	Low risk	Low risk	Low risk	Low risk	O

A. Study published only as an abstract. Insufficient details regarding randomization, allocation concealment, missing outcome data, Measurement of the outcome and selection of the reported outcome.

B. When analyzing the reported results for clinical pregnancy (137/193 *vs.* 124/193), we observed that the reported difference was not statistically significant p=0.19), but authors have claimed that it was significant (*p*<0.05), suggesting bias of the study group favoring the intervention.

C. Randomization was achieved by the embryologist according to a computer-generated randomization table a day before the embryo transfer. Both the clinician performing the embryo transfer and the patient were blinded for the allocation. Institutional review board approval was not obtained. Consecutive embryo transfer cycles (not participants) were randomized, allowing the same person to enter more than once in the study.

D. ET cycles (not participants) were randomized, allowing the same person to enter more than once in the study. Additionally, there is a substantial difference between the group size (N=155 in the study group and N=215 in the control group).

E. All patients meeting the study criteria were included after obtaining their consent. No patient refused to participate in the study. All patients included in the study proceeded to the stage of embryo transfer. Randomization to study or control group was performed on the day of embryo transfer based on a computer originated random number sequence, no details regarding allocation concealment. Outcomes were not defined.

F. Nondonor patients were randomized from a computer-generated random number sequence using sealed envelopes to either a treatment (ET with EmbryoGlue) or a control (ET with standard culture media) group. Clinical pregnancy was defined as at least one intrauterine gestational sac on ultrasound exam 2 weeks after positive beta hCG. A pregnancy was considered to be ongoing if fetal cardiac activity was present at 7 weeks of gestation. Data from live birth was obtained from the previously published review (Heymann *et al.*, 2022), but the results were close (49/117 vs. 39/108) to that reported for ongoing pregnancy (52/117 vs. 41/108). The study was not previously registered, and we did not find a published protocol. It is also not clear in the report whether the participants have signed informed consent.

G. The randomization to the study and control groups was performed using a computerized randomization table; however, there is no information regarding allocation concealment. It is not clear whether women were allowed to participate more than once in the study. Additionally, the proportion of women with previous implantation failure was larger in the control group (11/28=39% vs. 20/37=54%). The study was not previously registered, and we did not find a published protocol. It is also not clear in the report whether the participants have signed informed consent. Authors only presented “pregnancy rate” in their report; data for quantitative analysis were extracted from a previously published systematic review .

H. No information regarding randomization and allocation concealment. It is not clear whether women were allowed to participate more than once in the study. The study was not previously registered and we did not find a published protocol. It is also not clear in the report whether the participants have signed informed consent.

I. We identified only the registration in ClinicalTrials.gov. Study was terminated prematurely because the implantation rate was significantly lower in the treatment group using HA-Enriched medium for embryo transfer (https://clinicaltrials.gov/study/NCT00588250). No results were published online and we did not identify the final publication. Data for quantitative analysis were extracted from a previously published systematic review.

J. Some problems in their second randomization (Sellers *et al.*, 2022). No entire number would result in the reported proportions and the number of clinical pregnancies is lower than the number of live birth and miscarriages. We tried contact with the authors by several different emails, but we did not have any response.

K. No information regarding randomization and allocation concealment. It is not clear whether women were allowed to participate more than once in the study. The study was not previously registered and we did not find a published protocol.

L. As reported by the authors, “The study was conducted between June 2006 and June 2007. The last patient was recruited in February 2007.” However, the study was submitted to publication in Jan.29.2007. Patients were randomized according to a previously prepared computergenerated randomization list. Randomization was stratified for day 3 and day 5 transfers. The allocation was disclosed by the nurse coordinator to the chief embryologist who was loading the catheter, after the opening of consecutively numbered sealed opaque envelopes on the morning of the embryo transfer. The study group is the same of another included study (Balaban *et al.*, 2004) where the authors reported a significant difference following an intervention that is not compatible with the reported numbers.

M. Preliminary publication of a study (N=98) that planned to include a total of 250 women, but we did not identify the publication of the completed study.

N. The study group is the same of another included study (Balaban et al., 2004) where the authors reported a significant difference following an intervention that is not compatible with the reported numbers.

O. Trial Registration=NCT02725827, performed in two centers. One day before FET, the women were randomly assigned to either the HA group or the control group according to a computer-generated randomization list with a 1:1 ratio and a block size of 10. The randomization list was prepared by a designated research nurse who was not involved in patient care. The assignments were kept in sealed opaque envelopes, which were opened by the embryologist 1 day before FET to prepare the transfer medium. The embryologist was not otherwise involved in patient care. The women and the clinicians and nurses involved in recruitment, preparation, embryo transfer, and subsequent follow-up were all blinded to group assignments.

### Quantitative analysis

**Figure 2:**
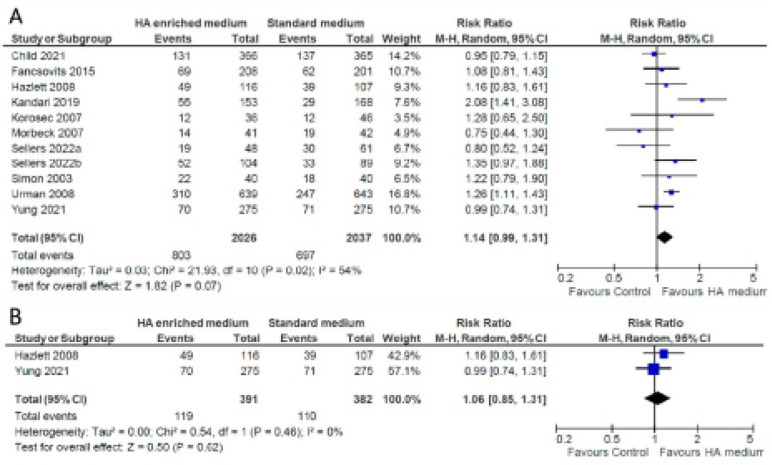
Results for live birth considering all the included studies (A) and only those not considered to be at high risk of bias (B).

The results for live births are presented in Figure 2a. A total of 11 studies were included in this analysis; the total number of participants allocated to the HA-enriched medium was 2,026 compared to 2,037 who were allocated to the standard transfer medium, encompassing 803 and 697 live births, respectively. The relative risk (RR) was 1.14 (95% CI=0.99-1.31), *p*=0.07. We observed a substantial heterogeneity, with I^2^=54%. Sensitivity analysis, including only the two studies not considered to be at high risk of bias (Figure 2b), resulted in a RR=1.06 (95% CI=0.85-1.31), *p*=0.62, with low heterogeneity (I^2^=0%). We did not use ongoing pregnancy as a surrogate outcome.

**Figure 3:**
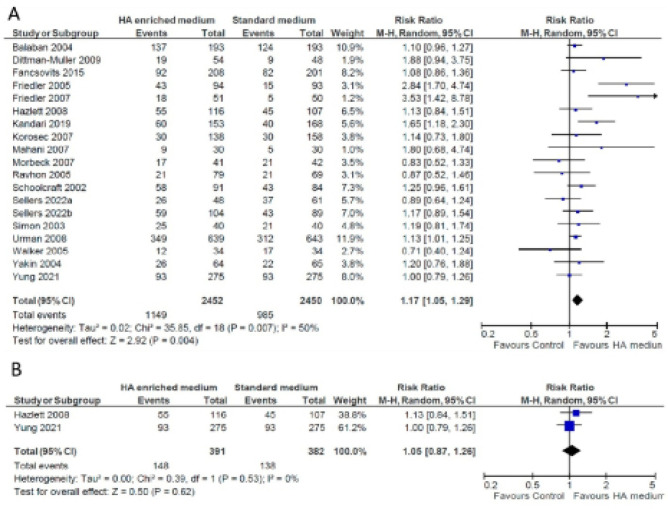
Results for clinical pregnancy considering all the included studies (A) and only those not considered to be at high risk of bias (B).

The results for clinical pregnancy are presented in Figure 3a. Only one study included in the quantitative analysis did not report clinical pregnancy but reported live birth (Child *et al.*, 2021). A total of 19 studies were included in this analysis; the total number of participants allocated to the HA-enriched medium was 2,452, compared to 2,450 who were allocated to the standard transfer medium, encompassing 1,149 and 985 clinical pregnancies, respectively. The RR was 1.17 (95% CI=1.05-1.29, *p*=0.004. We observed a substantial heterogeneity, with I^2^=50%. Sensitivity analysis, including only the two studies not considered to be at high risk of bias (Figure 3b), resulted in a RR=1.05 (95% CI=0.87-1.26), *p*=0.53, with low heterogeneity (I^2^=0%).

**Figure 4:**
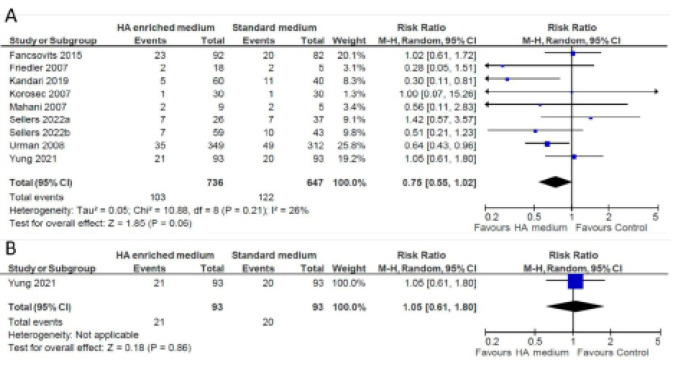
Results for pregnancy loss considering all the included studies (A) and only those not considered to be at high risk of bias (B).

The results for pregnancy loss are presented in Figure 4a. A total of 9 studies were included in this analysis; the total number of clinical pregnancies in participants allocated to the HA-enriched medium was 736, compared to 647 in the standard transfer medium, encompassing 103 and 122 pregnancy losses, respectively. The RR was 0.75 (95% CI=0.55-1.02, *p*=0.06. We observed a low heterogeneity, with I^2^=26%. Sensitivity analysis, including only one study not considered to be at high risk of bias (Figure 4b), resulted in a RR=1.05 (95% CI=0.61-1.80), *p*=0.86.

### Results from studies not included in the quantitative analysis

Four studies published solely as abstracts were not included in the quantitative analysis: (Chen *et al.*, 2001), (Drew *et al.*, 2014), Fasano *et al.* (2016), and Khan *et al.* (2004).

(Chen *et al.*, 2001): this study randomized 70 women. The observed results showed no significant difference for positive pregnancy test performed 14 days after the embryo transfer (8/35=23% *vs*. 5/35=14%, HA enriched medium *vs*. standard medium, respectively). The authors concluded that there is a trend towards a better pregnancy rate in an HA-enriched medium. Since this study reported only positive pregnancy tests, we could not extract data for the outcomes of interest.

(Drew *et al.*, 2014): this study randomized 493 embryo transfers. The observed results showed no significant difference for clinical pregnancy following either single embryo transfer (D3=30.2% *vs*. 23.3%, D5=44.1% *vs*. 42.4%, HA enriched medium *vs*. standard medium, respectively) or double embryo transfer (D3=30.4% *vs*. 39.7% and D5=44.4% *vs*. 42.4%). The authors concluded that using an HA-enriched medium resulted in no benefit to reproductive outcomes. We were not able to extract data for quantitative analysis because the total number of participants in each group was not reported.

Fasano *et al.* (2016): this study randomized 372 warming cycles from 253 women. Pregnancy rates per transfer were comparable between groups (29.4% *vs*. 23.9% *p*=0.32). Authors concluded that HA enriched medium is as effective as the standard medium. We were not able to extract data for quantitative analysis because authors reported the results for embryo transfer cycles, allowing the same participant to be included repeated times.

Khan *et al.* (2004): this study randomized 169 women aged below 39 years. The ongoing pregnancy rates were comparable between groups (53% *vs*. 50%). Authors concluded that HA enriched medium does not show any significant improvement when compared to standard medium. We were not able to extract data for quantitative analysis because the total number of participants in each group was not reported.

### Ongoing studies

Three ongoing studies were identified. One is being conducted in Abu Dhabi, United Arab Emirates (Nogueira, 2025). The study started on Jan. 2024; the authors estimated that the study will enroll 783 participants and will be completed by Dec. 2025.

The other ongoing study is being conducted in Xian, Shaanxi, China (Cai *et al.*, 2025), and it started on Oct. 2024. The authors estimated to enroll 858 participants and plan to complete the study by Sep. 2026.

Finally, there is a study been held in Wardha, India (Warhade *et al.*, 2025). There is an estimation to enroll 52 subjects, in the context of recurrent implantation failure. The predicted study completion is by Dec. 2026.

### Certainty assessment

The certainty of the evidence is reported in Table 5. The quality of the evidence for live birth, clinical pregnancy and pregnancy loss was judged to be of very low quality. Most of the studies were judged to be at high risk of bias, there was inconsistency among studies, and the estimates were not very precise. Moreover, although some of the published studies have found some benefit of the intervention, the only two studies that were not considered to be at high risk of bias did not show any effect, as well as the four studies that were not included in the quantitative analysis.

**Table 5:** Grade evidence profile for live birth, clinical pregnancy and pregnancy loss comparing hyaluronic acid enriched medium *vs*. standard 4 medium for embryo transfer

A.Comparative Summary of Outcomes and Overall Certainty												
Outcome	No. of Studies		HA Enriched Medium (n/N %)		Standard Medium (n/N %)		Relative Effect (RR, G5% CI)		Absolute Effect (per 1000)			Overall Certainty
Live birth	11		803/2026 (39.6%)		697/2037 (34.2%)		RR 1.14 (0.99 to 1.31)		48 more (from 3 fewer to 106 more)			⨁◯◯◯Very low a,b,c,d
Clinical Pregnancy	20		1157/2487 (46.5%)		990/2485 (39.8%)		RR 1.17 (1.06 to 1.29)		68 more (from 24 more to 116 more)			⨁◯◯◯Very low a,b,d
Pregnancy loss	9		103/736 (14.0%)		122/647 (18.9%)		RR 0.75 (0.55 to 1.02)		47 fewer (from 85 fewer to 4 more)			⨁◯◯◯Very low a,e
B. Details of Certainty Assessment (GRADE Factors)												
Outcome		Study Design		Risk of Bias		Inconsistency		Indirectness		Imprecision	Publication Bias	
Live birth		Randomised trials		Serious a		Serious b		not serious		Serious c	none	
Clinical Pregnancy		Randomised trials		Serious a		Serious b		not serious		Serious c	none	
Pregnancy loss		Randomised trials		Serious a		not serious		not serious		very serious d	none	

CI: confidence interval; RR: risk ratio; a: Serious risk of bias; b: Serious inconsistency; c: Serious imprecision; d: Very serious imprecision; e: Refers to the specific imprecision factor for pregnancy loss.

## DISCUSSION

This systematic review aimed to evaluate the effectiveness of using an HA-enriched medium for embryo transfer based on the currently available evidence. Due to the limitations of the included studies, inconsistency and imprecision, our level of certainty in the observed estimates is still very low for live births, clinical pregnancies, and pregnancy loss. The quantitative analysis considering all the included studies did not show a significant difference for live birth (RR=1.14, 95% CI=0.99-1.31) and pregnancy loss (RR=0.75, 95%CI=0.55-1.02), although there was a small increase in clinical pregnancies (RR=1.17, 95%CI=1.05-1.29). When analyzing only the two studies not considered to be at high risk of bias, none of the results were very similar for the live birth (RR=1.06, 95%CI=0.85-1.31), clinical pregnancy (RR=1.05, 95%CI=0.87-1.26), and pregnancy loss (RR=1.05, 95%CI=0.61-1.80). The four studies that were not included in the quantitative analysis also reported no benefit of the intervention.

Although the lists of included studies were very similar, there were some differences between this review and the previously published Cochrane review (Heymann *et al.*, 2022). Firstly, two studies (Ten *et al.*, 2019; Sellers *et al.*, 2022) were included that were not previously available. Additionally, data of clinical pregnancy were analyzed from Walker *et al.* (2005), that was included in the previous review, but authors reported that they failed to extract data because the study had reported the outcomes as percentage alone. However, authors have reported that there were 34 participants in each group, allowing to ultimately extract the data.

Moreover, the study of Fancsovits *et al.* (2011) was not included in our review because it comprises only a preliminary report of the complete study (Fancsovits *et al.*, 2015): the enrolment period reported by Fancsovits *et al.* (2011) was Jan. 2010 to Dec. 2010 while the enrolment period of Fancsovits *et al.* (2015) was Jan. 2010 to Aug. 2012. By including Fancsovits *et al.* (2011), the previous review counted the results for the same participant more than once, which is not appropriate.

In addition, we did not include one study where the comparison was not HA-enriched medium *vs*. standard medium for embryo transfer (Kleijkers *et al.*, 2016). In this study, participants were randomly assigned to have their oocytes and embryos cultured in one of the two media: G5 (Vitrolife, Goteborg, Sweden) and HTF (Lonza Verviers, Belgium). Although the G5 medium has HA in its composition and the HTF medium does not have HA, we do not believe the comparison is similar to using the HA-enriched medium only for embryo transfer. In this study, the authors compare two different standard media for the whole process, including embryo culture. Additionally, the concentration of HA in G5 (0.125 mg/mL) is very different from the HA-enriched medium for embryo transfer (0.5 mg/mL).

Finally, there is only one registered trial that will include only euploid blastocysts (19). It will be essential to follow up if the use of HA-enriched medium will influence treatment results within the group of euploid embryos.

Although the differences in the included studies were small, there is a crucial difference in the conclusions between the two reviews. In the previously published Cochrane review (Heymann *et al.*, 2022), authors have concluded that moderate-quality evidence shows improved clinical pregnancy and live birth rates with the addition of HA as an adherence compound in embryo transfer media. In the present review, we observed a very low level of certainty for the three outcomes, which were live birth, clinical pregnancy, and pregnancy loss. Moreover, based on the results of the only study judged to be at low risk of bias, we believe that there is no clinically relevant difference between using an HA-enriched medium or a standard medium for embryo transfer. Therefore, it is also suggested that we should wait for the results of the three extensive ongoing studies before making further recommendations for clinical practice and future research.

## References

[r1] Adeniyi T, Horne G, Ruane PT, Brison DR, Roberts SA. (2021). Clinical efficacy of hyaluronate-containing embryo transfer
medium in IVF/ICSI treatment cycles: a cohort study. Hum Reprod Open.

[r2] Balaban B, Urman B, Yakin K, Isiklar A, Kilic Y, Aksoy S. (2004). High pregnancy and implantation rates can be achieved in
blastocyst transfers using hyaluronan enriched culture and transfer
medium. Fertil Steril.

[r3] Balaban B, Yakin K, Ata B, Isiklar A, Urman B. (2011). Effect of hyaluronan-enriched transfer medium on take home baby
rate after day 3 and day 5 embryo transfers: a prospective randomized
study. Human Reprod.

[r4] Ben-Rafael Z, Ashkenazi J, Shelef M, Farhi J, Voliovitch I, Feldberg D, Orvieto R. (1995). The use of fibrin sealant in in vitro fertilization and embryo
transfer. Int J Fertil Menopausal Stud.

[r5] Braakhekke M, Kamphuis EI, Dancet EA, Mol F, Veen F van der, Mol BW. (2014). Ongoing pregnancy qualifies best as the primary outcome measure
of choice in trials in reproductive medicine: an opinion
paper. Fertil Steril.

[r6] Cai H, Xu D, Wang Z, Huang B, Xue X, Bai H, Man Y, Lei D, Wu Q, Ni Y, Lei J, Shi J. (2025). Efficacy and safety of hyaluronic acid-enriched transfer medium
in women undergoing single blastocyst transfer: a study protocol for a
multicentre randomised controlled trial. BMJ Open.

[r7] Chao S, Schenkman E, Kim S, Kenigsberg D, Brenner S, Moodie G. (2008). The effect of embryo glue on clinical pregnancy rate in frozen
embryo transfers. Fertil Steril.

[r8] Chen C, Tsai F, Lin J. (2001). The effect of combining human serum albumin and hyaluronic acid
in the transfer medium on the pregnancy rate in IVF cycles - a prospective
randomized study. Hum Reprod.

[r9] Child TJ, Bevan A, Frettsome-Hook R, Craig J, Shahbazian S, Mounce G. (2021). A randomised controlled blinded trial assessing the effectiveness
of embryoglue as an embryo transfer medium in ivf cycles. Fertil Steril.

[r10] Cowman MK, Schmidt TA, Raghavan P, Stecco A. (2015). Viscoelastic Properties of Hyaluronan in Physiological
Conditions. F1000Res.

[r11] Dittmann-Müller X, Zollner KP, Zollner U. (2009). Prospective randomised clinical trial about the efficacy of a
human embryo transfer medium (EmbryoGluew). Hum Reprod.

[r12] Drew C, Montgomery S, Campbell A, Fishel S. (2014). Prospective randomised controlled trial to assess the effect of
Embryoglue as a medium for embryo transfer. Hum Fertil.

[r13] Smeenk J, Wyns C, De Geyter C, Kupka M, Bergh C, Cuevas Saiz I, De Neubourg D, Rezabek K, Tandler-Schneider A, Rugescu I, Goossens V, European IVF Monitoring Consortium (EIM) for the European Society of
Human Reproduction and Embryology (ESHRE) (2023). ART in Europe, 2019: results generated from European registries
by ESHRE†. Hum Reprod.

[r14] Fancsovits P. (2014). Effect of Hyaluronan Enriched Embryo Transfer Media on IVF
Outcome.

[r15] Fancsovits P, Lehner A, Murber A, Kaszas Z, Rigo J, Urbancsek J. (2015). Effect of hyaluronan-enriched embryo transfer medium on IVF
outcome: a prospective randomized clinical trial. Arch Gynecol Obstet.

[r16] Fancsovits P, Murber A, Tothné Gilán Z, Rigó J, Urbancsek J. (2011). Effect of hyaluronan containing transfer media on pregnancy and
implantation rates in human IVF-ET cycles. A prospective randomized
study. Hum Reprod.

[r17] Fasano G, Antonacci R, Biramane J, Mbongolo G, Nguyen Thi M, Vanhelleputte C, Vannin A, Van Langendonckt A, Delbaere A, Devreker F. (2016). Clinical outcomes after use of Embryo-Glue as a human embryo
transfer (ET) medium in warming cycles. Hum Reprod.

[r18] Friedler S, Raziel A, Schachter M, Strassburger D, Kasterstein E, Komarovsky D, Bern O, Ron-El R. (2005). Efficacy of hyaluronan-enriched embryo transfer medium in
patients with repeated IVF-ET failures. Hum Reprod.

[r19] Friedler S, Schachter M, Strassburger D, Esther K, Ron El R, Raziel A. (2007). A randomized clinical trial comparing recombinant
hyaluronan/recombinant albumin versus human tubal fluid for cleavage stage
embryo transfer in patients with multiple IVF-embryo transfer
failure. Hum Reprod.

[r20] Hambiliki F, Ljunger E, Karlström PO, Stavreus-Evers A. (2010). Hyaluronan-enriched transfer medium in cleavage-stage
frozen-thawed embryo transfers increases implantation rate without
improvement of delivery rate. Fertil Steril.

[r21] Hazlett W, Meyer L, Karande V, Nasta T, Mangan P. (2004). Prospective, randomized, evaluation of the impact of using
embryoglue for the transfer medium in a non-selected group of
patients. Hum Reprod.

[r22] Hazlett WD, Meyer LR, Nasta TE, Mangan PA, Karande VC. (2008). Impact of EmbryoGlue as the embryo transfer
medium. Fertil Steril.

[r23] Heymann D, Vidal L, Or Y, Shoham Z. (2020). Hyaluronic acid in embryo transfer media for assisted
reproductive technologies. Cochrane Database Syst Rev.

[r24] Heymann D, Vidal L, Shoham Z, Kostova E, Showell M, Or Y. (2022). The effect of hyaluronic acid in embryo transfer media in donor
oocyte cycles and autologous oocyte cycles: a systematic review and
meta-analysis. Hum Reprod.

[r25] Higgins J, Savović J, Page M, Elbers R, Sterne J. Chapter 8: Assessing risk of bias in a randomized
trial. Cochrane Handbook for Systematic Reviews of Interventions version 65
2024; Cochrane.

[r26] Holt-Kentwell A, Ghosh J, Devall A, Coomarasamy A, Dhillon-Smith RK. (2022). Evaluating interventions and adjuncts to optimize pregnancy
outcomes in subfertile women: an overview review. Hum Reprod Update.

[r27] Kandari S. (2019). Time lapse selected elective single embryo transfer in hyaluronon
enriched transfer medium in PCOS improves live birth rates compared to use
of conventional embryo transfer media. A possible alternative to freeze-all
cycles in PCOS. Fertil Steril.

[r28] Karimian L, Rezazadeh Valojerdi M, Baghestani AR, Moeini A. (2004). A prospective randomized comparison of two commercial embryo
transfer medium in IVF/ICSI cycles. Hum Reprod.

[r29] Khan I, Urich M, Sasy M, Shmoury M, Abuzeid MAyers J. (2004). A prospective, randomized study comparing EmbryoGlue and P1
Complete as embryo transfer medium. Hum Reprod.

[r30] Kleijkers SH, Mantikou E, Slappendel E, Consten D, van Echten-Arends J, Wetzels AM, van Wely M, Smits LJ, van Montfoort AP, Repping S, Dumoulin JC, Mastenbroek S. (2016). Influence of embryo culture medium (G5 and HTF) on pregnancy and
perinatal outcome after IVF: a multicenter RCT. Hum Reprod.

[r31] Korosec S, Virant-Klun I, Tomazevic T, Zech NH, Meden-Vrtovec H. (2007). Single fresh and frozen-thawed blastocyst transfer using
hyaluronan-rich transfer medium. Reprod Biomed Online.

[r32] Lensen S, Hammarberg K, Polyakov A, Wilkinson J, Whyte S, Peate M, Hickey M. (2021). How common is add-on use and how do patients decide whether to
use them? A national survey of IVF patients. Hum Reprod.

[r33] Mahani IM, Davar R. (2007). Hyaluronic acid versus albumin in human embryo transfer
medium. East Mediterr Health J.

[r34] Marei WFA, Wathes DC, Raheem KA, Mohey-Elsaeed O, Ghafari F, Fouladi-Nashta AA. (2017). Influence of hyaluronan on endometrial receptivity and embryo
attachment in sheep. Reprod Fertil Dev.

[r35] Morbeck DE. (2007). Prospective Randomized Clinical Trial of Novel Implantation Promoting
Medium (EmbryoGlue) to Improve IVF Success Rates.

[r36] Nakagawa K, Horikawa T, Orita Y, Yamashiro E, Watanabe H, Shirai A, Ogata S, Kataoka H, Kuroda K, Takamizawa S, Sugiyama R. (2023). Hyaluronan-enriched transfer medium (HETM) can improve the
implantation rate in morphologically poor euploid blastocyst
transfer. Arch Gynecol Obstet.

[r37] Nakagawa K, Takahashi C, Nishi Y, Jyuen H, Sugiyama R, Kuribayashi Y, Sugiyama R. (2012). Hyaluronan-enriched transfer medium improves outcome in patients
with multiple embryo transfer failures. J Assist Reprod Genet.

[r38] Nishihara T, Morimoto Y. (2017). Evaluation of transfer media containing different concentrations
of hyaluronan for human in vitro fertilization. Reprod Med Biol.

[r39] Nogueira D. (2025). Impact of Hyaluronan-enriched Medium on Pregnancy Outcomes Following
Euploid Blastocyst Transfers.

[r40] Perez O, Adriaanse H, Tilley B, Navarrete G, Lay L, Little LM, Gada R, Lawrence L, Lee K, Thomas MR, Chantilis S. (2019). The effect of extended blastocyst exposure of hyaluronan enriched
transfer media on implantation rate in frozen embryo
transfers. Fertil Steril.

[r41] Ravhon A, Nahum H, Weissman A, Biran G, Umansky N, Levran D. (2005). Embryo Transfer in Hyaluronan Enriched Transfer Medium Does Not
Improve Pregnancy Rate in IVF Treatment. Fertil Steril.

[r42] Rodriguez-Martinez H, Tienthai P, Atikuzzaman M, Vicente-Carrillo A, Rubér M, Alvarez-Rodriguez M. (2016). The ubiquitous hyaluronan: Functionally implicated in the
oviduct?. Theriogenology.

[r43] Schoolcraft W, Lane M, Stevens J, Gardner DK. (2002). Increased hyaluronan concentration in the embryo transfer medium
results in a significant increase in human embryo implantation
rate. Fertil Steril.

[r44] Schünemann HJ, Brennan S, Akl EA, Hultcrantz M, Alonso-Coello P, Xia J, Davoli M, Rojas MX, Meerpohl JJ, Flottorp S, Guyatt G, Mustafa RA, Langendam M, Dahm P. (2023). The development methods of official GRADE articles and
requirements for claiming the use of GRADE - A statement by the GRADE
guidance group. J Clin Epidemiol.

[r45] Sellers R, Ten J, Arnedo AR, Guerrero J, Moliner B, Llácer J, Bernabeu R. (2022). The use of Embryoglue® (EG) for embryo transfer does not
improve live birth rates: a prospective randomized controlled
trial. Reprod Biomed Online.

[r46] Simon A, Safran A, Revel A, Aizenman E, Reubinoff B, Porat-Katz A, Lewin A, Laufer N. (2003). Hyaluronic acid can successfully replace albumin as the sole
macromolecule in a human embryo transfer medium. Fertil Steril.

[r47] Ten J, Sellers R, Rodríguez-Arnedo A, Guerrero J, Ortiz JA, Moliner B, Bernabeu R. (2019). Embryoglue® as medium for embryo transfer: does it really
improve the outcomes? A prospective randomized controlled
trial. Hum Reprod.

[r48] Thornton M, Ghaemi S, Keskintepe L. (2018). Does the utilization of embryo glue give promise in donor embryo
fets?. Fertil Steril.

[r49] Tomari H, Honjou K, Kunitake K, Nishimura K, Hidaka N, Nagata Y. (2014). Effect of embryo glue transfer medium during fresh and
frozen-thawed embryo transfer. Fertil Steril.

[r50] Tyler B, Walford H, Tamblyn J, Keay SD, Mavrelos D, Yasmin E, Al Wattar BH. (2022). Interventions to optimize embryo transfer in women undergoing
assisted conception: a comprehensive systematic review and
meta-analyses. Hum Reprod Update.

[r51] Urman B, Yakin K, Ata B, Isiklar A, Balaban B. (2008). Effect of hyaluronan-enriched transfer medium on implantation and
pregnancy rates after day 3 and day 5 embryo transfers: a prospective
randomized study. Fertil Steril.

[r52] Valojerdi MR, Karimian L, Yazdi PE, Gilani MAS, Madani T, Baghestani AR. (2006). Efficacy of a human embryo transfer medium: a prospective,
randomized clinical trial study. J Assist Reprod Genet.

[r53] Walker DL, Thornhill AR, Allemand MC, Tatpati LL, Wentworth MA, Tummon IS. (2005). A Randomized, Controlled, Double Blinded Trial of
EmbryoGlue® in Frozen Embryo Transfer Cycles: An Interim
Analysis. Fertil Steril.

[r54] Warhade A, More A, Choudhary N, Kalaskar G, Warhade P. (2025). Association of Hyaluronic Acid-Enriched Transfer Medium (HETM)
with Clinical Pregnancy Outcome in Women Undergoing Frozen-Thawed Embryo
Transfer (FET). J Pharm Bioallied Sci.

[r55] Yakin K, Balaban B, Isiklar A, Bozdag H, Urman B. (2001). Improved clinical outcome in frozen-thawed embryo transfers with
the use of hyaluronan-enriched transfer medium. Hum Reprod.

[r56] Yung S, Lai SF, Lam MT, Lui MW, Ko JKY, Li HWR, Lau EYL, Yeung WSB, Ng EHY. (2019). Hyaluronan-enriched embryo transfer medium for frozen-thawed
embryo transfer: a double-blind randomised controlled trial. Hum Reprod.

[r57] Yung SSF. (2016). Randomized Controlled Trial Comparing Hyaluronan-enriched Embryo
Transfer Medium Versus Control for Frozen-thawed Embryo Transfer.

[r58] Yung SSF, Lai SF, Lam MT, Lui EMW, Ko JKY, Li HWR, Wong JYY, Lau EYL, Yeung WSB, Ng EHY. (2021). Hyaluronic acid-enriched transfer medium for frozen embryo
transfer: a randomized, double-blind, controlled trial. Fertil Steril.

